# Response to Wilson *et al.* Comments on Lopez-Jaramillo *et al.* DivinylSulfone Cross-Linked Cyclodextrin-Based Polymeric Materials: Synthesis and Applications as Sorbents and Encapsulating Agents. *Molecules*, 2015, *20*, 3565–3581.

**DOI:** 10.3390/molecules21010098

**Published:** 2016-01-15

**Authors:** Francisco Javier Lopez-Jaramillo, Fernando Hernández-Mateo, Francisco Santoyo-Gonzalez

**Affiliations:** Departamento de Química Orgánica, Facultad de Ciencias, Instituto de Biotecnología, Universidad de Granada, Granada E1871, Spain; fhmateo@ugr.es

Wilson *et al.* raisea number of issues that, according to their opinion, require explanation. They support their comment on their estimation of the accessibility of β-cyclodextrin (β-CD) sites of four polymers prepared by cross-linking β-CD with divinylsulfone (DVS) at four stoichiometries. In particular, they focus their attentions on our conclusion that for our system (*i.e.*, seven polymers and six sorbates) the degree of cross-linking “plays a minor role on the formation of the inclusion complexes” [sic] and extend their criticism to the isotherms, the content of cross-linker and the limited molecular characterization. Before addressing each specific issue, it is important to remark that the polymers reported by Wilson *et al.* in their comments are neither characterized nor the degree of cross-linking is estimated experimentally. Singularly, they report the β-CD:DVS ratio used for the synthesis as the only estimation of cross-linking degree and the statement “the materials were synthesized according to the verbatim reported method” (*i.e.*, our paper) as the description of the synthesis. Providing that Wilson *et al.* performed the synthesis correctly, and that their polymers have a different degree of cross-linking, it is important to recall the warning recently published by these authors in Microporous Mesoporous Mater. [1], where the comparison of the effect of the mode of addition of the cross-linker led them to conclude: “Therefore, precautions should be taken when comparing materials from independent studies since synthetic conditions under thermodynamic *vs.* kinetic control may result in products with variable structure and textural properties” [sic].

Wilson’s data are incomplete and difficult to evaluate and the comparison with our results is not straightforward. Their data seem to be a particular case since they particularize their analysis on the inclusion complexes of a single model compound (*i.e.*, phenolphthalein) with four homopolymers of β-CD, whereas our conclusion was based on six different compounds (*i.e.*, phenol, 4-nitrophenol, β-naphthol, bisphenol A, progesterone and curcumin) and seven different polymers, comprising homopolymers and heteropolymers, but only a single case of β-CD homopolymer as those synthesized by Wilson *et al.* No less important is the fact that three of the four polymers studied by Wilson *et al.* were prepared with “cross-linking ratios at much lower levels” (*i.e.*, DVS:β-CD stoichiometries at the synthesis step) and that “heteropolymers containing starch were not examined since such materials are less amenable to the phenolphthalein dye based method” [sic].

Despite the limitations of Wilson’s study, it arises a number of points that we would like to clarify in this reply.

## 1. Questioning of the High Content of DVS Preparation

The aim of our work was the synthesis of insoluble polymeric materials and their evaluation as sorbents. From a practical point of view the yield and the reproducibility of the synthesis are important parameters to take into account for the use of these materials in real problems. Preliminary experiments revealed that low DVS:carbohydrate ratios led to very low yields that compromised the practical interest of the synthesis. Conversely, high ratios yielded polymers that tended to become aggregated and yielded inhomogeneous material. Hence we focused on the stoichiometries reported in our paper. The cross-linker incorporation was evaluated by elemental analysis as the S/C ratio since the only source of sulfur was DVS and, as expected, we found a clear relationship between sulfur content and the amount of DVS employed (Table 1 of our paper). It is important to be aware that this relationship is not straightforward and that use the DVS:β-CD ratio as the parameter to discuss the degree of cross-linking is not reliable.

## 2. Influence of the Cross-Linking on the Formation of Inclusion Complexes

**Figure 1:**
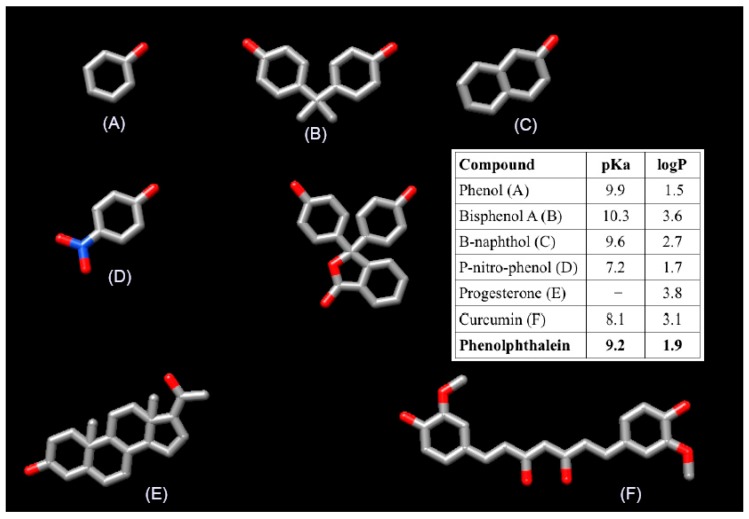
Structure and the features of the five compound (**A**–**F**) studied in our paper and the phenolphthalein used by Wilson *et al.*

As discussed above, the scope of Wilson’s study is limited since it focuses on homopolymers of β-cyclodextrin with an estimated degree of cross-linking lower that ours. The materials that we reported in our paper were two homopolymers of starch, one homopolymer of β-CD, two homopolymer of α-CD, and two heteropolymers of β-CD/starch and α-CD/starch, respectively. Additionally, we evaluated six different sorbates that are very different to phenolphthalein in terms of size, structure and polarity (Figure 1). In this context it is not unexpected that their results may differ from ours. It is important to recall that our conclusion was: “The degree of cross-linking exerts a clear influence on the surface of the material, whereas it plays a minor role on the formation of the inclusion complexes” [sic] and that this is supported by our experimental data.

## 3. Limited Molecular Level Characterization of the Cross-Linked Material

Our polymers were characterized by ATR-IR, powder X-ray, SEM, TGA and elemental analysis. The use of phenolphthalein to estimate the accessibility of β-CD is old known. However, for our system it is not suitable since as Wilson *et al.* stated in their comment “heteropolymers containing starch were not examined since such materials are less amenable to the phenolphthalein dye based method” [sic]. On the other hand, from the three remaining polymers, two of them were synthesized by cross-linking α-CD, the polymer obtained cross-linking β-CD being the only material amenable for the phenolphthalein assay. In this context, the contribution of a single point experiment to question the seven polymers reported in our paper should be taken with precautions. Instead of phenolphthalein, we used phenol and *p*-nitrophenol as model compounds because, besides the importance of phenolic compounds as toxic pollutants commonly encountered in trace quantities in aqueous effluents, the thermodynamic parameters of their inclusion in cyclodextrin have been reported [2] and there exists bibliography that exploits the inclusion of phenolic compounds in cyclodextrins [3,4,5,6,7,8].

## 4. Reliability of the Linearized Adsorption Models

We are aware of the misuse of linearization and the abuse of R^2^ in model comparison [9]. We focused on the linearized forms of the Freudlich and Langmuir isotherms (unweighted fit) because they are widely used. Moreover, according to Foo and Hameed [10] despite the bias “linearization remains a confident option in literature, applied to over 95% for the liquid-phase adsorption systems”. In this context and since both Langmuir and Freundlich isotherms are two parameter isotherms, R^2^ has been used to evaluate the fitting of the experimental data to the linearized isotherm forms. This approach is shared by other articles recently published in Molecules. For example in a paper published in 2014 [11] the authors use the linearized form of the isotherm and state: “The Freundlich plot has a lower correlation coefficient than the Langmuir plot. This suggests that the use of the Freundlich isotherm is limited. The Langmuir model effectively describes the equilibrium sorption data (Figure 9); the linear plot has a good correlation coefficient (>0.99) and the maximum adsorption capacity is close to that determined experimentally” [sic]. Additionally, the presentation of the isotherm in the linearized manner is also accepted in other journals [12].

Regarding the use of co-solvents, it is common knowledge that it influences the results and in fact this was reported in our paper for the sorption of β-naphthol. At this point it is important to recall that the translation to real problems of the parameters estimated from the formation of inclusion complexes with the model molecule phenolphthalein at pH 10.5 and 0.04% (*v*/*v*) ethanol is not straightforward. On the other hand, the use of co-solvents to yield the formation of inclusion complexes is accepted in the pharmaceutical industry and in order to remove pollutants from the water, the presence of other substances may be the real concern. Hence, the presence co-solvents does not invalidate our results as long as their concentration and nature are well defined, as it is in the case of our paper. 
